# Endogenous Retroviral–K Envelope Is a Novel Tumor Antigen and Prognostic Indicator of Renal Cell Carcinoma

**DOI:** 10.3389/fonc.2021.657187

**Published:** 2021-04-22

**Authors:** Veronika Weyerer, Pamela L. Strissel, Christine Stöhr, Markus Eckstein, Sven Wach, Helge Taubert, Lisa Brandl, Carol I. Geppert, Bernd Wullich, Holger Cynis, Matthias W. Beckmann, Barbara Seliger, Arndt Hartmann, Reiner Strick

**Affiliations:** ^1^ Institute of Pathology, University Hospital Erlangen, Friedrich-Alexander University Erlangen-Nuernberg, Erlangen, Germany; ^2^ Department of Gynecology and Obstetrics, University Hospital Erlangen, Comprehensive Cancer Center, European Metropolitan Area Erlangen-Nuremberg (CCC ER-EMN), Friedrich-Alexander-University Erlangen-Nuernberg, Erlangen, Germany; ^3^ Adjunct Affiliation With Department of Radiation Oncology, University of Maryland School of Medicine, Baltimore, MD, United States; ^4^ Department of Urology and Pediatric Urology, University Hospital Erlangen, Friedrich-Alexander University Erlangen-Nuernberg, Erlangen, Germany; ^5^ Department of Drug Design and Target Validation, Fraunhofer Institute for Cell Therapy and Immunology, Halle, Germany; ^6^ Institute of Medical Immunology, Martin Luther University Halle-Wittenberg, Halle, Germany; ^7^ Translational Research Centre (TRC), Erlangen, Germany

**Keywords:** endogenous retrovirus, ERV-K, renal cell carcinoma, tumor antigen, patient prognosis, p53, azacytidine, invasion

## Abstract

Renal cell carcinoma (RCC) is one of the ten most common cancers for men and women with an approximate 75% overall 5-year survival. Sixteen histological tumor subtypes exist and the most common are papillary, chromophobe and clear cell renal cell carcinoma (ccRCC) representing 85% of all RCC. Although epigenetically silenced, endogenous retroviral (ERV) genes become activated in tumors and function to ignite immune responses. Research has intensified to understand ERV protein function and their role as tumor antigens and targets for cancer (immune) therapy. ERV-K env is overexpressed and implicated as a therapeutic target for breast cancer, however studies in RCC are limited. In this investigation a human RCC tissue microarray (TMA) (n=374) predominantly consisting of the most common histological tumor subtypes was hybridized with an ERV-K env antibody and correlated with patient clinical data. TMA results showed the highest amount of ERV-K env protein expression and the strongest significant membrane expression in ccRCC versus other RCC subtypes. High ERV-K env total protein expression of all tumor subtypes significantly correlated with low tumor grading and a longer disease specific survival using multivariable analyses. Cell proliferation and invasion were assayed using the kidney cell lines HEK293 with wild-type p53 and a ccRCC cell line MZ1257RC mutated for p53. Transfecting these cell lines with a codon optimized *ERV-K113 env* overexpressing CMV vector was performed with or without 5’-Aza-2’-deoxycytidine (Aza) treatment to sustain promoter de-methylation. MZ1257RC showed induction of *ERV-K113* expression and significantly increased both proliferation and invasion in the presence or absence of Aza. HEK293 cells demonstrated a restriction of *ERV-K113 env* expression and invasion with no changes in proliferation in the absence of Aza. However, in the presence of Aza despite increased *ERV-K113 env* expression, an inhibition of HEK293 proliferation and a further restriction of invasion was found. This study supports ERV-K env as a single prognostic indicator for better survival of RCC, which we propose represents a new tumor antigen. In addition, ERV-K env significantly regulates proliferation and invasion depending on p53 status and Aza treatment.

## Introduction

ERVs and related sequences are estimated at ~400,000 copies or 4.6% of the human genome, including genomic proviruses like 5’ long terminal repeat (LTR)-gag-pol-env-3’ LTR (1). On the other hand, LTR-retrotransposons, so called mammalian apparent LTR retrotransposons (MaLRs) represent 3.6% of the genome and lack primer binding sites and gag-pol genes (1). Due to recombination events between 5’ and 3’ LTRs, throughout evolution internal genes were lost resulting in ERV solitary LTRs (solo-LTRs). These solo-LTRs contain functional sequences like promoters, enhancers, polyadenylation signals and are frequently located near cellular genes. Specifically, ERV-K members were originally identified due to their similarity to the Mouse Mammary Tumor Virus (MMTV) and are subdivided into ten so-called human MMTV-like clades (HML1–10) (2, 3). It is estimated for ERV-K HML1 through HML10 that ~600 proviruses and ~4,400 solo-LTRs exist (4, 5). Underestimated are polymorphisms of *ERVs* within the global human populations like *ERV-K*, which can be used to distinguish Africans, Europeans and East Asians (6). Especially *ERV-K113* and *ERV-K115* have been assessed with allele frequencies between 21-34% in individuals from Africa and only 1-4% in the United Kingdom (7).

Epigenetic *ERV* activation occurs in a broad spectrum of tumors and functions at the RNA level to ignite an interferon (IFN) immune response *via* dsRNA formation coined “viral mimicry” (8). At the protein level, ERVs can drive hormone induced tumor growth and anchorage independent growth (9), cancer cell-cell fusion (9, 10), regulation of immune cells, and mediate signal transduction pathways (8, 11). Overexpressed ERVs can also serve as tumor-self antigens (12). *ERV-K* represents one of the largest and most active *ERV*-groups, as well as one of the most recently integrated *ERVs* (*ERV-K* HML2) with multiple copies of intact ORFs (4, 13). In addition to the ERV-K env protein two alternative splice products Rec or Np9 are also actively involved in carcinogenesis (14). Like other *ERVs*, *ERV-K* is silenced by various epigenetic processes (15). However, DNA hypomethylation can activate *ERV-K* during normal human embryogenesis until the late blastocyst, but also in different cancer types, like melanoma and breast cancer (14, 16, 17). Treating breast cancer cell lines and human breast tumor xenografts with ERV-K specific antibodies resulted in decreased tumor growth and apoptosis supporting ERV-K env is a tumor antigen which can be targeted for therapy (18).

The *ERV-E* family consists of more than 1,300 elements subdivided into LTR2, LTR2B and LTR2C (19–21). Of these elements only a few were proviruses, like *ERV-E* at chromosome 6q15 and *ERV-E 4-1* at chromosome 19p12. However, neither of the latter coded for a full length env (e.g. *ERV-E6q15* env and *ERV-E4-1* env codes for ORFs of 211 and 427 amino acids, respectively). Several ERVs, especially *ERV-E 6q15*, have been found activated in renal cell cancer (RCC) and correlated with an immunotherapy response (20, 22–25). Specifically, the *ERV-E6q15* provirus encodes a highly immunogenic 10 amino acid (aa) peptide considered as a tumor-specific antigen (22). Interestingly, this 10 aa peptide was detected in the blood of a patient with RCC following an allogeneic hematopoietic stem cell transplantation, which led to patient tumor cell killing *in vitro* (22).

RCC is among the ten most common cancers for both men and women with an approximate 76% overall survival (OS) of 5 years, but only 12% of patients with metastatic stage IV disease (26). An increased risk for RCC includes smoking (3-fold), obesity, hypertension, diet and alcohol. Additionally, older age, genetic predisposition syndromes such as Von Hippel-Lindau (VHL) disease as well as male gender also predispose for RCC (26, 27). Histological subtypes include clear cell renal cell carcinomas (ccRCC), which comprise 75-80% of all kidney tumors, as well as papillary, chromophobe and others. One hallmark of ccRCCs are deletions of chromosome 3p, which eminently result in the loss of the *VHL* tumor suppressor gene (27, 28). *VHL* gene mutations but also hypermethylation of the *VHL* promoter contribute to attenuating its tumor suppressor mechanisms.

Research has intensified to understand ERV protein function as well as their role as tumor antigens being targets for cancer immune therapy. In this investigation we focused on ERV-K env RNA and protein expression and function in RCC as well as the importance of ERV-K env for prognosis of patients with RCC.

## Materials and Methods

### RCC Tissue Microarray, Pathological and Patient Clinical Results

The present study was approved by the Ethics Commission of the FA-University of Erlangen-Nuernberg (# 3755). The procedures were performed in accordance with the ethical standards established in the 1964 Declaration of Helsinki and later amendments. All patients gave written consent for the use of their tumor material. We implemented an RCC TMA, which contained 453 tissues derived from formalin-fixed and paraffin-embedded tumors as previously described (29). The resection date ranged from 1998 to 2011. The TMA was reviewed by three Uropathologists (VW, ME, AH) to verify tumor histology according to the latest WHO classification of 2016 (30). All tumor tissues of the TMA represented regions from the tumor center. Among the 453 cases we excluded 79 cases due to tumor absence from multiple tissue block usages overtime. Therefore, for this present study we analyzed 374 tumors, of different RCC subtypes including ccRCC (n= 288), papillary (type I (n= 18); type II (n= 27), chromophobe (n= 27), ccRCC with sarcomatoid features (n= 8), and others (n= 6). Patient survival data was updated in 2019 with follow-up data available for 314 patients (84%), with a median potential follow-up time of 65.5 months. Pathological and clinical parameters and follow-up patient survival data in months are shown in **Supplementary Table 1**.

### Immunohistochemistry

Hybridization of a purified rabbit polyclonal antibody specific for ERV-K env (MyBioSource Cat. #: MBS9216561; 1: 1,000 dilution) was performed with the RCC TMA or hybridized with MZ1257RC and HEK293 tissue cytoblocks transfected with a codon optimized overexpressing *ERV-K113* env gene cloned into a pcDNA3.1-vector with a CMV promoter or an empty control pcDNA3.1vector (see transfections below). The ERV-K env peptides representing the antigen specific for the antibody are shown in **Supplementary Table 2**. IHC was performed with the mouse & rabbit specific HRP/DAB detection kit according to manufacturer’s protocol (Abcam). The ERV-K env positive tumors in the TMA were classified according to localization (membranous and cytosolic) and intensities scored as H-scores. The H-score represents the sum of all intensities scored as percentages [0 {negative}, 1+ {weak}, 2+ {moderate} and 3+ {strong} (31)], which are then combined to a score capped at 300. The H-Score has no dimension. H-Score-Formula: H-score = {1 × (% cells 1+) + 2 × (% cells 2+) + 3 × (% cells 3+)}. Every H-score of the RCC TMA is represented in **Supplementary Table 3**. Additionally, p53 (Dako, clone: DO-7, monoclonal mouse Anti-Human antibody, dilution 1: 50) immunohistochemistry was performed on a BenchMark ULTRA Automated IHC/ISH Slide Staining System (Ventana; Ventana Medical Systems, Inc., Tucson, AZ, USA). IHC positive p53 cells were either defined as strong nuclear overexpression in at least 10% of cells or with a complete p53 loss of expression (‘null phenotype’).

### Cell Lines and Cell Culture Experiments

Eleven ccRCC cell lines (MZ1257RC, Caki1RC, MZ1790RC, MZ1795RC, MZ2861RC, MZ1846RC, MZ2733RC, MZ1851RC, MZ1774RC, MZ1973RC, MZ2905RC) were previously established from primary ccRCC and cultured according to Seliger et al. (32). The MZ1257RC cell line was used in all functional studies and cultured in DMEM media, 10% FCS and L-Glutamine. The human embryonic kidney cell line HEK293 was cultured in DMEM F12 media and 10% FCS. All cell lines were cultured at 37°C at 5% CO_2_.

### RNA Isolation and Gene Expression Studies of Primary Tissues and Cell Lines

RNA isolation of microdissected primary ccRCC tissues (n= 14) and available patient matched tumor associated normal renal tissues (n= 11) was performed using an automated magnetic bead-based system (Maxwell^®^ 16 Instrument and modified using the RSC DNA blood kit along with the RNA incubation and lysis buffer from Promega). For RNA isolation of cell lines Trizol (Life Technologies) was used according to manufacturer’s instructions. All RNAs were treated with DNase I and quantified. Real time PCR was performed using a StepOnePlus and SYBR^®^-Green (Thermo Fisher) detection according to (8). Specific primers were used to quantify seven codogenic *ERV* gene families (*ERV-W, ERV-H, ERV-Fc1, ERV-Fc2, ERV-T, ERV-K, ERV-E*) using absolute quantitative real time PCR converted to absolute molecules/ng RNA as previously described (33). Primers for three reference genes, *18S-rRNA*, TF 5’ GCAATTATTCCCCATGAACG, BR 5’ GGCCTCACTAAACCATCCAA; *ß-actin*, TF 5’ TCACCATTGGCAATGAGCGG, BR 5’ GATGTCCACGTCACACTTCAT; and *RPS23*, TF 5’ TGGAGGTGCTTCTCATGCAAA, BR 5’ TAATGGCAGAATTTGGCTGTTTG were used for normalization according to MIQE guidelines (34). Additionally, note that primers specifically detecting the transfected codon optimized *ERV-K113* env gene did not hybridize with the endogenous *ERV-K env* gene (35).

### Transfection and 5’-Aza-2’deoxycytidineTreatment of Cells

HEK293 and MZ1257RC cells were seeded at 450,000 or 250,000 cells per 95 mm^2^ tissue culture dish, respectively, and the next day transfected either with 3 µg of the codon optimized overexpressing vector with a *ERV-K113 env* gene (35) or with the control pcDNA3.1 vector (Invitrogen) using JetPei (Polyplus). A *pEGFP-N1* vector (Clontech) (3 µg) was transfected and confirmed a transfection efficiency >80% for both cell lines. RNAs were harvested post transfection at 16 h, 24 h, 48 h and 72 h in the presence or absence of the DNA methyltransferase (DNMT) inhibitor Aza (500 nM) (Sigma) to establish a time kinetic of expression according to Chiappinelli et al. (8) (n= 2). Tissue cytoblocks were made following transfection with the codon optimized overexpressing vector with a *ERV-K113 env* gene or the control pcDNA3.1 vector at 16 h. Briefly, transfected cells were detached with 0.0625% Trypsin (Gibco) and cell pellets fixed with 4% PFA for 1 h. The pellet was washed with 1x PBS, stained with 100% Eosin for 5 min, washed with 1x PBS and then embedded in Histogel (Thermo Scientific) overnight at 4°C. Following formalin fixation, ethanol dehydration and paraffin treatment overnight at 60°C, the tissue culture pellets were embedded in paraffin at 4°C.

### Proliferation and 3D Collagen Invasion Assays

Following transfection with the codon optimized overexpressing vector containing an *ERV-K113 env* gene or with the pcDNA3.1 control vector (after 16 h), 2 x 10^5^ MZ1257RC or HEK293 cells were seeded in 95 mm^2^ culture dishes to assay for proliferation in the presence or absence of Aza. The total number of MZ1257RC and HEK293 cells transfected with the overexpressing *vector* containing the codon optimized *ERV-K113 env* gene or with the pcDNA3.1 control vector were determined using a Neubauer hemocytometer chamber at 44 h, 68 h and 92 h post transfection (n= 4 for MZ1257RC; n= 2 for HEK293). In parallel, 3D collagen invasion assays were initiated with 50,000 transfected MZ1257RC (n= 6) and HEK293 cells (n= 3) in the presence and absence of Aza according to Weigand et al. (36). Invasion scores represented as invaded cells/mm^2^ were determined at 68 h post transfection.

### Statistical Analysis

Descriptive statistics were employed to characterize the distributions of continuous as well as nominal variables. Non-parametric Wilcoxon rank-sum test was used for comparison of continuous variables and partitioning testing (mono-forest prediction) was performed to determine “cut-off” levels for each tumor subtype because of known biological differences between the histological RCC tumor subtypes. Multivariable systems were used and variables included for models were the following: gender, pT-Stage, pN-stage, lympho-vascular and angiogenic invasion, presence of distant metastasis, tumor grading and morphology. All p-values were two-sided and a p-value <0.05 was considered statistically significant. All statistical analyses were performed using GraphPad Prism 7.2 (GraphPad Software Inc.) and JMP SAS 15.2 (SAS).

## Results

### Gene Expression of ERV Env Genes in ccRCC and Normal Primary Renal Tissues

**Figure 1:**
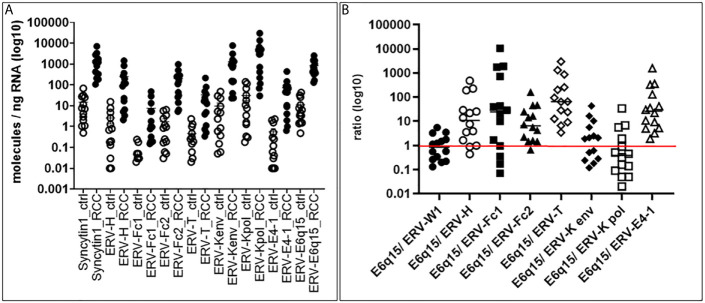
*ERV* gene expression in primary tissues. **(A)** *ERV* expression in ccRCC and control kidney tissues. Seven different *ERV* gene families were analyzed for expression in molecules/ng RNA (log10) (Y-axis) from 14 primary ccRCC and 11 patient matched tumor associated control tissues (X-axis). Gene expression was then compared using Mann-Whitney two-tailed test, where all ccRCC tumors were significantly increased compared to controls (p < 0.0001). **(B)** ERV6q15 env expression ratios in primary ccRCC. The graph shows for each ccRCC (n= 14; same tissues as in A) the specific *ERV* expression from 7 families (A) in a ratio (log10) (Y-axis) compared with *ERV-E6q15* (X-axis - E6q15).

In order to examine the expression of codogenic ERV families, we quantified the mRNA expression levels of seven *ERV* gene families using 14 primary ccRCC and 11 patient matched tumor associated normal kidney tissues (**Figure 1A**). Compared to normal kidney samples, all *ERV* genes were significantly higher expressed in the ccRCC lesions (p < 0.0001) (**Figure 1A**). For example, *ERV-K env* was 134.6-fold higher expressed in ccRCC compared to normal kidney (1,281.33 vs 9.52 molecules/ng RNA). The most well studied *ERV* gene in ccRCC is *ERV-E6q15*, where high transcript levels have been noted (20, 25). We found that *ERV-E6q15* env was 76.29-fold higher expressed in ccRCC compared to controls (832.43 vs 10.91 molecules/ng RNA). Examining the ratio of *ERV-E6q15 env* with other *ERV* genes revealed a higher expression of *ERV-E6q15 env* when compared to the env genes of *ERV-E4-1, ERV-H, ERV-T, ERV-Fc1* and *ERV-Fc2* (**Figure 1B**). In contrast, *ERV-W1 env* (*Syncytin-1*) and *ERV-K env* were higher expressed in approximately 50% of ccRCC compared to *ERV-E6q15 env*. Interestingly, *ERV-K pol* had the highest expression in >50% of RCC compared to *ERV-E6q15 env* (**Figure 1B**). The findings that other *ERV* genes are highly expressed in ccRCC where transcript levels exceed *ERV-E6q15 env* support a functional role for these *ERV* genes in RCC.

### ERV-K Env Protein Localization in Primary RCC Tissues and Clinical Associations

**Figure 2:**
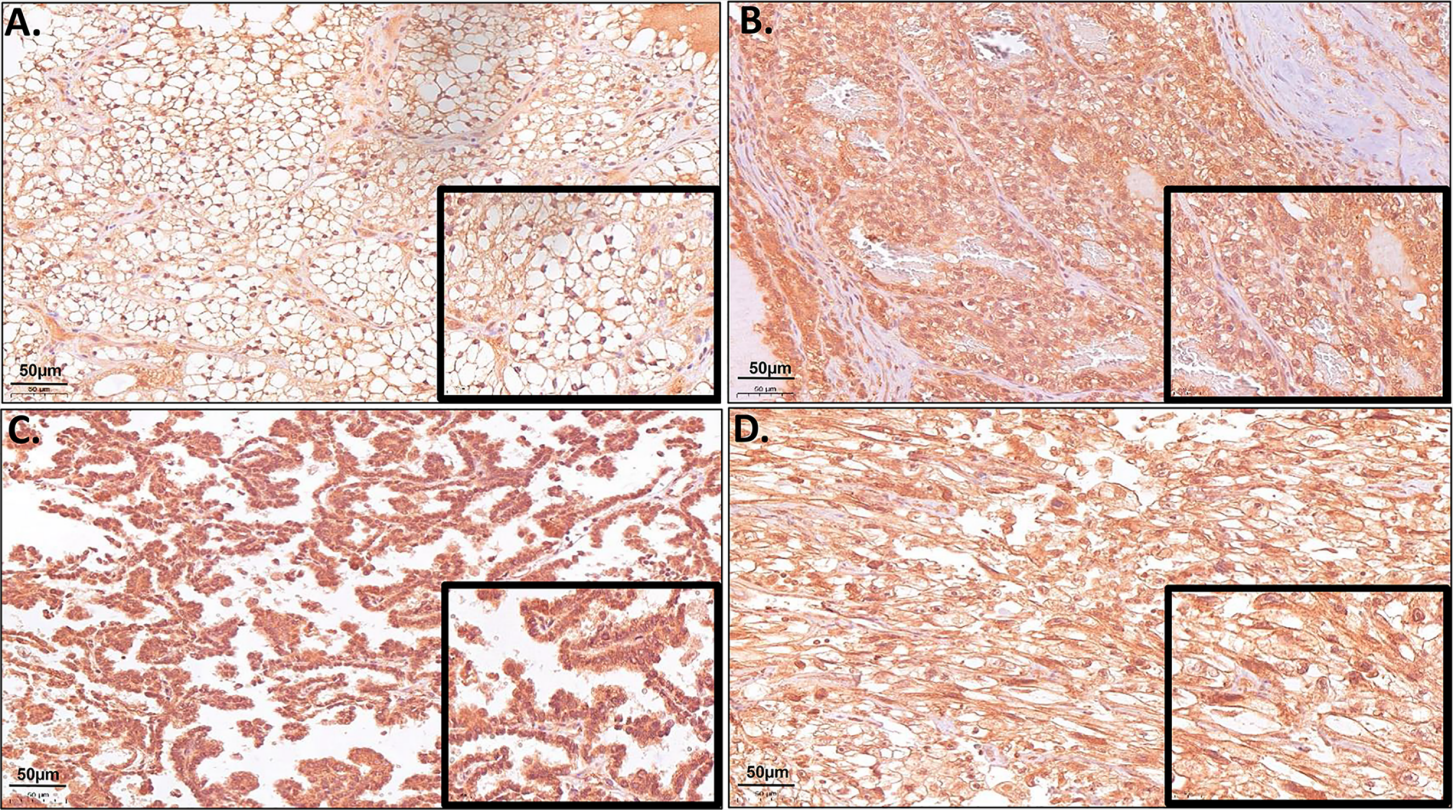
Representative microscopic images of TMA samples of different RCC tumor subtypes hybridized with a specific ERV-K env antibody. **(A)** Example of a ccRCC (Tumor #21 TMA I) with strong protein expression of ERV-K env at the cell membrane. **(B)** Example of a ccRCC (Tumor #23 TMA I) with strong cytosolic enrichment for ERV-K env protein. **(C)** Example of a RCC papillary tumor with strong cytosolic enrichment for ERV-K env protein (Tumor #28 TMA II). **(D)** Example of a ccRCC with sarcomatoid features (Tumor #20 TMA III) with strong membranous as well as a partly cytosolic ERV-K env staining. Bar indicates 50µm.

**Figure 3:**
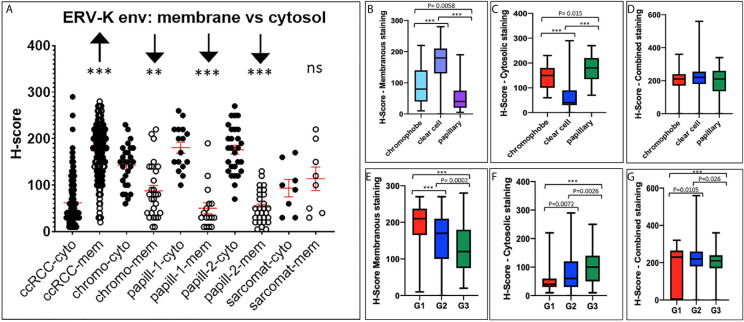
ERV-K env protein localization and clinical correlations between RCC tumor subtypes. **(A)** ERV-K env cytosolic (cyto) or membrane (mem) localization represented as H-scores (Y-axis; 0 - 400) for each RCC tumor TMA subtype (X-axis). TMA; ccRCC = clear cell carcinoma (n= 288) with sarcomatoid (Sarcomat) features (n= 8); Chromo = chromophobe (n= 27); papill-1 and -2 = Papillary 1 (n= 18) and 2 (n= 27). ***p< 0.0001; **p= 0.0003, ns = not significant. Red bar crosses represent the mean for each cohort tumor subtype. **(B)** Bar graphs comparing ERV-K env membranous or **(C)** cytosolic staining or **(D)** total protein expression (combined) with the H-score significance and RCC tumor subtypes. **(E)** Bar graphs comparing ERV-K env membranous or **(F)** cytosolic or **(G)** total protein expression (combined) for each RCC tumor subtype staining with the H-score significance comparing RCC tumor subtypes and tumor grading (G1, G2, G3). All p-values are indicated within the graphs or ***p< 0.001.

Due to the high gene expression of *ERV-K env* in ccRCC (**Figure 1**) and the availability of specific antibodies, a TMA consisting of 374 RCC tissues was implemented to determine the ERV-K env protein expression and localization within cancer cells (**Figure 2**). Analyzing the amino acid sequence used as the antigen for the ERV-K env rabbit polyclonal antibody in this study, we identified at least 15 different members of ERV-K env at specific chromosomal regions, which might contribute to the overall ERV-K env expression (**Supplementary Table 2**). The H-score of ERV-K env showed a variable distribution and intensity of membrane vs cytosolic expression among all tumors (**Figures 2**, **3**). ERV-K env demonstrated the strongest expression of ccRCC at the membranes compared to the cytosol (p< 0.0001) (**Figures 2A**, **3A** and **Supplementary Table 3**). In contrast, all other tumor subtypes exhibited stronger cytosolic ERV-K env expression compared to cell membranes (papillary type I and II, p< 0.0001; chromophobe, p= 0.0003) (**Figures 2C**, **3A**). For ccRCC with sarcomatoid features, we observed that ERV-K env expression was equally distributed between the membrane and cytosol (**Figures 2D**, **3A**). Finally an evaluation between tumor subtypes demonstrated that ccRCC had the strongest significant ERV-K env membrane expression compared to papillary and chromophobe subtypes (**Figures 3B, C**).

**Figure 4:**
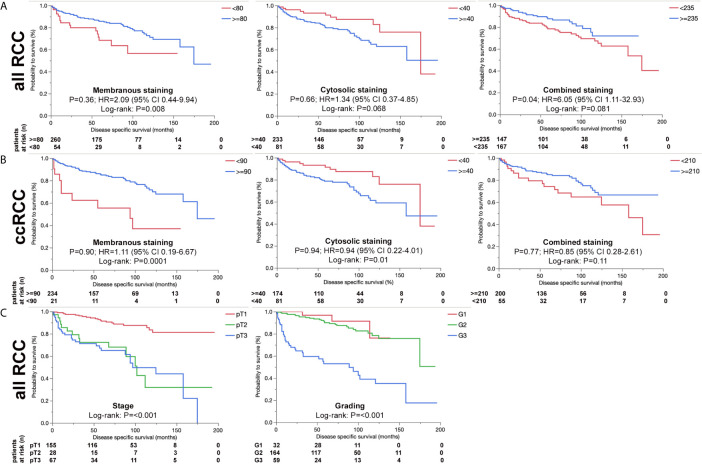
ERV-K env and associations with RCC patient survival. **(A)** Kaplan-Meier survival curves {Disease specific survival (DSS) in months} of all RCC patients as represented from the TMA showing the association of ERV-K env membranous, cytosolic and total protein expression (combined) according to the H-score (> blue or < red). Note that ERV-K env total protein shows a significance for longer DSS survival. **(B)** Kaplan-Meier survival curves (Disease specific survival in months) of ccRCC patients represented from the TMA showing the association of ERV-K env membranous, cytosolic and total protein expression (combined). **(C)** Kaplan-Meier survival curves of all RCC patients represented from the TMA according to staging and grading. For staging the following regressions were: pT2 vs pT1: HR 13.7; p= 0.0002; 95%-CI: 3.39 - 55.33; pT3 vs pT1: HR 6.36; p= 0.03; 95%-CI: 1.17 - 34.71; pT3 vs pT2: HR 0.46; p= 0.32; 95%-CI: 0.1 – 2.08. For Grading the regressions were: G3 vs G2 + G1: HR 2.28; p= 0.23; 95%-CI: 0.6 - 8.71. For all curves log rank p-values are shown as well as multivariable adjusted Hazards ratios (HR) calculated by using Cox regression models. Partitioning tests (mono-forest prediction) were performed to determine “cut-off” levels for each tumor subtype because of known biological differences between the histological RCC tumor subtypes.

We further analyzed ERV-K-env protein expression for correlations with patient clinical parameters. For all RCC subtypes tumor grading demonstrated differences according to the localization of ERV-K env expression (**Figure 3**). G1 tumors exhibited the highest significant ERV-K env expression and G3 tumors the lowest at the membrane, but was reciprocal in the cytosol (**Figures 3E, F**). Analyzing pT-staging no significant correlations with ERV-K env expression were observed (data not shown). For all RCC subtypes strong expression levels of total cellular ERV-K env protein significantly correlated with low tumor grading (**Figure 3G**) and a longer disease specific survival (DSS) using a multivariable analysis (p= 0.04, HR= 6.05, 95% CI: 1.11 – 32.93) (**Figure 4A**). This finding supports that both ERV-K env membrane and cytosol protein expression are important for patient clinical outcome. Analyzing single tumor subtypes no significant correlations of ERV-K env expression with DSS were found (**Figure 4B** for ccRCC). As proof of concept for all RCC subtypes grading and staging correlated significantly with DSS (**Figure 4C**).

### ERV Gene Expression in ccRCC and HEK293 Cell Lines

**Figure 5:**
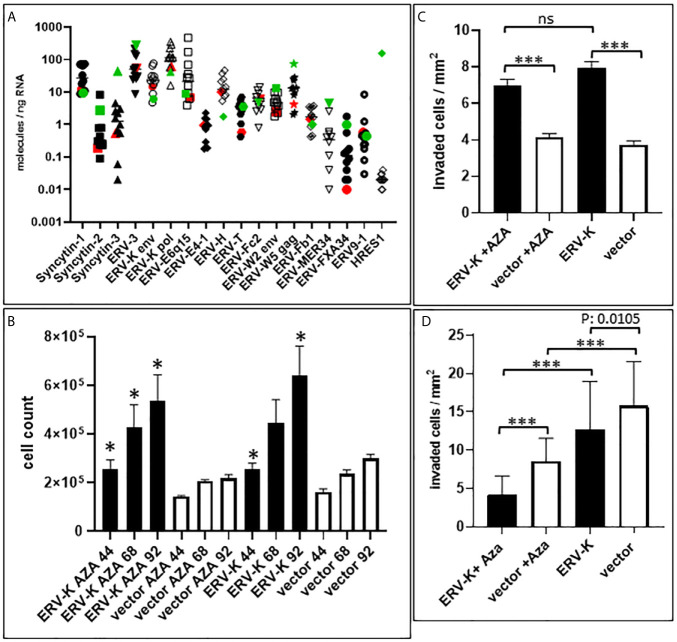
*ERV* gene expression in ccRCC cell lines and functional studies. **(A)** Graph shows gene expression (molecules/ng RNA, Y-axis) profiling 16 different *ERV* gene families (X-axis) of 12 different cell lines (ccRCC = 11 see *Materials and Methods*; Red= MZ1257RC ccRCC cell line; Green = HEK293). Note that *ERV-Fc1 env* and *ERV-Rb env* are not shown on the graph since they were undetectable for gene expression. **(B)** Graph shows MZ1257RC cell proliferation (Y-axis = total cell counts) following transient transfection with the overexpressing CMV vector containing a codon optimized *ERV-K113 env* gene at 44 h, 68 h and 92 h post transfection in the presence or absence of Aza. (n= 4); *p = 0.0286. **(C)** Graph shows MZ1257RC 3D cell invasion into collagen (Y-axis = # of invaded cells/mm^2^) following transient transfection with the overexpressing CMV vector containing a codon optimized *ERV-K113 env* gene at 68 h post transfection in the presence or absence of Aza. (n= 6), ***p < 0.0001; ns = non-significant. **(D)** Graph shows HEK293 3D invasion into collagen (Y-axis = # of invaded cells/mm^2^) following transient transfection with the overexpressing CMV vector containing a codon optimized *ERV-K113 env* gene at 68 h post transfection in the presence or absence of Aza. (n= 3), ***p < 0.0001.

**Figure 6:**
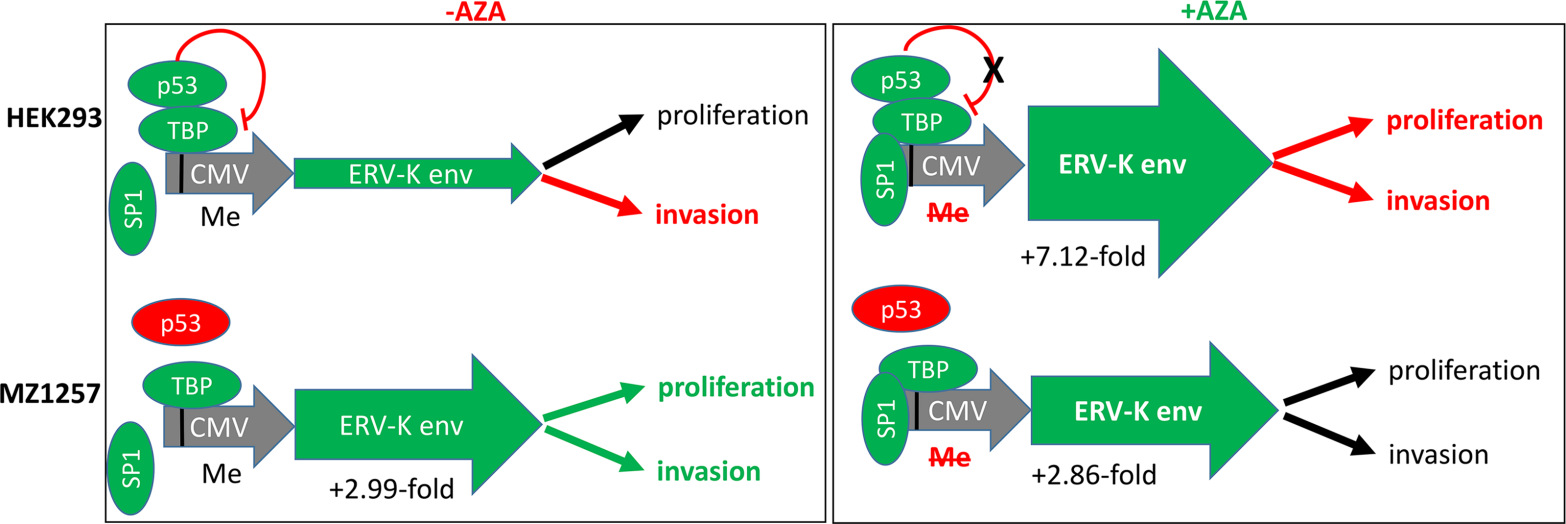
Results and model showing transcriptional regulations and cellular functions of HEK293 and MZ1257RC cell lines following *ERV-K113 env* gene transfection in the presence or absence of Aza treatment. Left schematic shows top HEK293 cells or bottom MZ1257RC cells transfected with the overexpressing CMV vector containing a codon optimized *ERV-K113 env* gene in the absence of Aza (-Aza). The CMV promoter (grey) with p53 binding to TATA binding protein (TBP) (38) is methylated (Me). Note that the transcription factor SP1 is not bound. The p53 wt protein is a repressor (green) for HEK293 cells, but mutant (dysfunctional) p53 (red) for the MZ1257RC cell line. For HEK293 cells the green arrow (ERV-K env) indicates less expression, when compared with MZ1257RC cells. A 2.99-fold increase of *ERV-K113 env* gene expression for MZ1257RC (2^-ΔΔCt^ = -Aza = 3,113.2) is indicated compared to HEK293 cells (2^-ΔΔCt^ = -Aza = 1,038.5) at 24 h post transfection. Right schematic shows top HEK293 cells or bottom MZ1257RC cells transfected with the overexpressing CMV vector containing a codon optimized *ERV-K113 env* gene in the presence of Aza (+Aza). P53, TBP and SP1 (40) are bound at a de-methylated CMV promoter (grey) promoting transcription. In the presence of Aza an inhibition of DNA-methylation (Me) and a lack of p53 repression is predicted for HEK293 cells. For both cell lines *ERV-K113 env* green arrows indicate higher levels of expression when compared to –Aza treated cells (left schematic). *ERV-K113* env gene expression for HEK293 was 7.12-fold higher (2^-ΔΔCt^ = +Aza = 7398.7) and for MZ1257RC cells a 2.86-fold induction (2^-ΔΔCt^ = +Aza = 8927.69) at 24 h. Note that for both cell lines the 2^-ΔΔCt^ *ERV-K113 env* gene expression levels were similar at 24 h after Aza treatments. To the far right for both cell lines functional outcomes are indicated for proliferation (at 44 h, 68 h, 92 h) and invasion (68 h), when compared to in the presence or absence of Aza treatment (see **Figure 5**, **Supplementary Figure 2**) (black arrows = no change; red arrows = inhibition; green arrows = increase).

The profile of *ERV* gene expression from 16 different families was analyzed with 11 ccRCC cell lines as well as with HEK293 cells. *ERV-W1 env* (*Syncytin-1*), *ERV-W5 gag, ERV-3, ERV-K env* and *pol*, *ERV-E6q15*, and *ERV-H env* were the highest expressed genes in these cell lines (**Figure 5A**). In contrast, *HRES-1* (HTLV-related endogenous sequence), an ERV expressing a gag-like protein (37) was the lowest expressed in ccRCC cells, but the highest in HEK293 cells (**Figure 5A**). To determine the protein function of ERV-K env stemming from an overexpressing vector, among all the cell lines we chose MZ1257RC and HEK293 expressing comparable low gene expression levels of endogenous ERV-K env (**Figure 5A**). In addition, HEK293 demonstrated p53 wild type (wt) expression and localization, whereas MZ1257RC was determined as mutant with a p53 localization solely in nuclei (**Supplementary Figure 1**). Transfection experiments with a CMV based codon optimized *ERV-K113 env* overexpressing vector demonstrated the highest gene and protein expression levels after 16 h and 24 h post-transfection, which then rapidly decreased by 48 h (**Supplementary Figure 1** and data not shown). It is known that p53 wt transcriptionally represses the CMV promoter *via* binding to the transcriptional machinery (38) and that cells can generally methylate and silence transfected vectors (39). Therefore, we compared gene expression levels of the codon optimized *ERV-K113 env* gene between both cell lines with opposite p53 genotypes in the presence or absence of Aza at 24 h post transfection. In the absence of Aza, HEK293 cells showed ~3-fold less *ERV-K113 env* gene expression compared to MZ1257RC, supporting p53 repressor activity in HEK293 cells, but a loss of repression in MZ1257RC (**Figure 6**). We propose this result is due to the p53 mutant protein in the MZ1257RC cell line. Interestingly, Aza treatment of both cell lines increased and sustained transfected *ERV-K113 env* gene expression until 72 h to almost similar levels (**Figure 6**).

### Influence of ERV-K113 Env Overexpression on Cell Proliferation and 3D Invasion

Following transfection of MZ1257RC cells with the codon optimized *ERV-K113 env* overexpressing vector, proliferation was significantly induced from 44 to 92 h post-transfection with or without Aza treatment when compared with cells transfected with the control vector (**Figure 5B**). However, we noted no significant difference of proliferation of *ERV-K113 env* transfected cells between the presence or absence of Aza treatment. In contrast, HEK293 cells showed no induction of proliferation following transfection with *ERV-K113 env*, however, interestingly in the presence of Aza, proliferation was restricted even in the presence of *ERV-K113 env* (**Supplementary Figure 2**). When MZ1257RC cells were transfected with the overexpressing vector containing the codon optimized *ERV-K113 env* gene and treated with or without Aza, we observed a significantly higher 3D cell invasion at 68 h post-transfection compared to cells transfected with the control vector (**Figure 5C**). On the other hand, cell invasion was inhibited with HEK293 cells transfected with the *ERV-K113 env* gene overexpressing vector, but interestingly a further inhibition was observed in the presence of Aza (**Figure 5D**). In order to explain the converse proliferation and invasion results between MZ1257RC and HEK293 upon Aza treatment, the p53 protein status is pivotal. As previously shown a lack of an Aza response was noted with other mutant p53 cancer cell lines (41). Taken together overexpression of ERV-K env regulates tumor cell proliferation and invasion, however a p53 mutant status could explain the lack of suppression of proliferation and invasion following Aza treatment of MZ1257RC cells (**Figure 6**).

## Discussion

Tumor antigens are categorized into different classes. One class reflects gene mutations within tumor cells, which are translated and processed into neoantigens (42). These neoantigens are then presented to T-cells *via* the major histocompatibility complex (MHC) and initiate tumor cell killing. Another class of tumor antigens represent overexpressed proteins (tumor-self antigens) (42). Ideal criteria for tumor antigens as targets for therapy are enhanced immunogenicity associated with an anti-tumor response, which directly suppresses an oncogenic phenotype essential to the tumor. Since ERVs are epigenetically silenced in somatic cells, activation and overexpression is an opportunity to study their function in tumors. In addition, targeting overexpressed ERV proteins in tumors could represent a therapeutic approach resulting in a better patient clinical outcome. Our present investigation along with the literature brings forth new results supporting ERV-K env as a possible tumor-self antigen in RCC involved in proliferation and invasion.

Our initial gene expression profiling of 11 *ERV* families not only showed significantly overexpressed levels for these *ERV* genes in ccRCC compared to normal kidney tissues, but also identified several *ERV* genes, which were in part higher expressed compared to the ccRCC associated bona fide *ERV-E6q15 env* gene (22, 25). Among these, *ERV-W1 env* (*Syncytin-1*), *ERV-Fc1 env, ERV-K env* and *pol* were higher expressed than *ERV-E6q15*, which led us to focus on *ERV-K env* for further studies. Interestingly, except for *ERV-Fc1* we found that these same *ERV* genes were also expressed in 11 different ccRCC cell lines. Although, our present study was limited to ccRCC cell lines, there is a need to analyze *ERV* gene expression in non-ccRCC tumor cell lines. However, only a few RCC papillary and no chromophobe cell lines exist to date (43).

Although, *ERV-E6q15 env* is considered an important ccRCC tumor antigen, presently there is no protein data available localizing its expression within tumor cells. Our detailed assessment of ERV-K env protein expression in RCC tissues showed that membrane and cytosolic localization were significantly different between RCC subtypes supporting functional roles of ERV-K env at both cellular compartments. Furthermore, the ERV-K env antibody used in the present study has the potential to hybridize with more than 15 ERV-K env members sharing the same or near identical antigen binding site. This finding supports amplified ERV-K env tumor functions. Lastly, RCC ERV-K env protein localization in its entirety significantly correlated with low tumor grading and a longer patient DSS, thus supporting ERV-K env as a prognostic indicator for patient survival.

In the realm of oncoimmunology immune cell infiltrates found in RCC (44), or other tumors, like bladder (45), breast (46), and colon (47) correlated with patient survival. Current checkpoint inhibition therapies show promising effects in RCC patients by inhibiting PD-L1 or CTLA-4 pathways (44). In the last years, epigenetic regulation and activation of *ERV* RNA, especially dsRNA, induced an IFN mediated innate immune response (8) and further activated cellular immunity in mouse models (48, 49). A global bioinformatics approach profiling ERVs (hervQuant) of different human tumors using The Cancer Genome Atlas (TCGA) RNA expression database revealed that *ERV* gene expression significantly associated with clinical prognosis of ccRCC patients (23). *ERV* expression in ccRCC also correlated significantly with a B-cell activation response. As specific predictors for immune checkpoint therapy remain uncertain in ccRCC, interestingly a recent study demonstrated an association of response with the expression of the putative codogenic *ERV3-2* gene (50). Another TCGA bioinformatics profiling of 18 different cancers demonstrated *ERV-E* and *7* different *ERV-K* families specifically associated with ccRCC (51). In addition, ERV-E env could be a potential tumor-restricting target for T-cell based immunotherapy (25).

In different cancers a loss of epigenetic silencing transcriptionally activated *ERVs* and resulted in adaptive immune responses to ERV epitopes (52–54). Spontaneous T-cell and B-cell responses against ERV antigens are well documented (55, 56). Examples from patients with melanoma or RCC, specific ERV antigens were recognized by T-cells with potent anti-tumor activity, demonstrating ERV proteins as important targets for immune based elimination (22, 57). Particularly, ERVs, like ERV-K are part of the cancer testis antigen group, exclusively expressed in germ cells and testis, but also in multiple cancers (58, 59). ERV-K env protein expression was specifically expressed in breast tumor tissues and cell lines, elicited a B-cell response and increased antibody titers in a large proportion of patients as well as mediated T-cell anti-tumor killing (31, 60).

Oncogenic phenotypes in association with *ERV-K env* expression were noted in our study as well as in previous investigations (11, 18, 61). Importantly, using specific primers only detected the transfected codon optimized *ERV-K113 env* gene and not the endogenous gene, where levels were low in both HEK293 and MZ1257RC cells. Therefore, we attributed functional changes of tumor cells due to ERV-K113 env expression. In addition, we observed that the p53 status may also play an essential functional role. The ~3-fold restriction of *ERV-K113 env* gene expression of p53 wt HEK293 cells compared to p53 mutant MZ1257RC cells in the absence of Aza, supports that p53 wt functions as a repressor of the CMV promoter (**Figure 6**). In addition, HEK293 and MZ1257RC cells transfected with the *ERV-K113 env* gene showed opposite functional phenotypes for proliferation and invasion, where HEK293 cells possibly overcompensated high ERV-K113 env protein levels restricting invasion, whereas in MZ1257RC the restriction was lost. One possible contributing mechanism of restriction may stem from translational processing occurring in the Golgi or ER, which was shown previously for HEK293 cells upon transfection with the same codon optimized overexpressing vector containing the *ERV-K113 env* gene (35). Important signaling pathways identified in the literature could explain ERV-K113 env protein regulation of proliferation and invasion. Analyzing human breast cancer cell lines *in vitro* and in mice following overexpression or knockdown of the *ERV-K env* gene led to an increase or decrease, respectively, of proliferation, invasion and metastasis (11). The latter demonstrated that p53, RAS/RAF/MEK/ERK protein signaling were involved in regulating these tumor functions. In another study, overexpression of the *ERV-K env* gene in human cell lines activated ERK1/2 signaling, induced an epithelial to mesenchymal transition (EMT) and increased invasion (61).

In addition to DNA demethylation *via* DNMT inhibition, Aza treatment of cells increased p53, activation of p21 and a cell cycle arrest due to DNA damage (62, 63). TP53 is an important tumor suppressor gene frequently mutated in human cancers (64). In our study, Aza treatment of both cell lines increased and sustained *ERV-K113 env* gene expression. Aza inhibited HEK293 cell proliferation with a further decrease of invasion supporting an induction of the p53 wt pathway despite activation of *ERV-K113 env* gene expression. In the presence or absence of Aza, MZ1257RC cell proliferation and invasion did not change, supporting these tumor processes are driven by ERV-K113 env protein and the p53 status as already shown with other cancer cell lines (41, 63, 65). P53 wt exerts repression of the transcriptional machinery at the CMV promoter (**Figure 6**) (38). On the other hand p53 wt activates transcription at *LTRs* following treatment with DNA damaging agents, however, activation was low or non-responsive with mutant p53 (66). In our cell culture studies the CMV over expressing vector was a tool to unravel ERV-K113 env protein function in p53 wt and mutant cells. Interestingly, when we examined 16 different endogenous *ERV* gene family expression levels, we noted that expression was higher in p53 wt HEK293 compared to mutant p53 MZ1257RC cells (**Figure 5A**). Although, we have not analyzed the LTRs from these codogenic *ERV* gene families for p53 binding sites, we support the idea that p53 is a transcriptional activator of *ERV* gene expression *via* LTRs (66), depending on the epigenetic landscape (67), but represses *ERV* gene expression in the absence of p53 binding sites by interfering with other proteins (68).

Taken together, evidence from the literature and our present study show that *ERV-K env* members; I) are significantly higher expressed in RCC compared to normal kidney tissue; II) are accessible at the membrane for possible antibody targeting, especially in ccRCC; III) strong expression levels of total cellular ERV-K env protein is associated with a better RCC patient clinical outcome and finally our functional findings show that; IV) ERV-K113 env modulates cancer cell proliferation and invasion depending on DNA methylation and correlates with the p53 status supporting ERV-K env represents a tumor-self antigen.

## Data Availability Statement

The datasets presented in this study can be found in online repositories. The names of the repository/repositories and accession number(s) can be found in the article/**Supplementary Material**.

## Ethics Statement

The studies involving human participants were reviewed and approved by Ethic Commission of the FA-University of Erlangen-Nuernberg, Germany #3755. The patients/participants provided their written informed consent to participate in this study.

## Author Contributions

RS and PS conceived the project, wrote the paper and performed functional analysis. VW analyzed the TMA, correlated the clinical patient data and wrote the paper. CS and AH provided the TMA and microdissected primary tissues. VW, ME, and AH analyzed the TMA. SW, HT and BW provided clinical data. HC provided the *ERV-K113 env* clone. LB helped with TMA IHC, technical assistance and formulated ideas. CG was responsible for the digital scanning of the TMA. MB assisted to write the paper. BS provided the RCC cell lines and helped with editing the manuscript. All authors contributed to the article and approved the submitted version.

## Funding

The present study was funded from a general scientific pool of funds from each participating Institution.

## Conflict of Interest

The authors declare that the research was conducted in the absence of any commercial or financial relationships that could be construed as a potential conflict of interest.

## Supplementary Material

Supplementary Figure 1 Cytoblock of MZ1257RC and HEK293 cell sections hybridized with p53 and the ERV-K113 env antibody. Top left (HEK293) and right (MZ1257RC) photos show p53 localization hybridizing with a specific p53 antibody to cells harvested at 16 h post transfection with the overexpressing CMV vector containing a codon optimized *ERV-K113 env* gene. No difference was found with the untransfected control pcDNA3.1 vector (not shown). Middle panel left (HEK293) and right (MZ1257RC) photos show ERV-K113 env protein cellular localization hybridizing with a specific ERV-K113 env antibody to cells harvested at 16 hours post *ERV-K113 env* CMV vector transfection. Below left (HEK293) and right (MZ1257RC) photos show hybridization with a specific ERV-K113 env antibody to cells harvested at 16 hours post transfection with the control pcDNA3.1 CMV vector. A time gene expression kinetic showed the highest expression at 16 h (HEK293 2^-ΔΔCt^ = 31,028.66; n= 2; MZ1257RC 2^-ΔΔCt^ = 106,902.78; n= 2). Note that at 16 h the p53 wt HEK293 cell line was 3.44-fold lower in *ERV-K113 env* gene expression compared to the p53 mutant MZ1257RC cell line. This result was similar comparing both cell lines at the 24 h kinetic time point shown in **Figure 6**.

Supplementary Figure 2 HEK293 cell proliferation. Graph shows HEK293 cell proliferation (Y-axis = total cell counts) following transient transfection with the overexpressing CMV vector containing a codon optimized *ERV-K113 env* gene at 44 h, 68 h and 92 h post transfection in the presence or absence of Aza. (n= 2).

Supplementary Table 1 Clinical-pathological parameters of the RCC cohort. This table summarizes the main pathological as well as the clinical characteristics of the analyzed renal cell tumors. Left column represents the histological subtypes, pN-stage, pM-stage, pT-stage, lymphovascular invasion, blood vessel invasion, WHO/ISUP grading, gender, patient age and available survival follow-up data of patients; middle column represents the actual number of available tumors or the number of available patients for follow-up survival data and the median potential follow-up time in months; and to the right the percentage of analyzed tumors compared to the total available tumors and patients available for follow-up of survival data.

Supplementary Table 2 Antigens of 15 ERV-K env members specific for the ERV-K env antibody used in this investigation. This table shows 15 different ERV-K env gene members with the antigen binding site specific for hybridization with the ERV-K env antibody used in this study. The first column shows the *ERV-K env* gene members localized at specific chromosomal regions; the second column shows the antibody binding site to the antigen which spans 32 amino acids; and the third column shows the gene accession number (access. nr).

Supplementary Table 3 IHC ERV-K env H-scores of RCC TMA (n= 374). This table shows all membrane staining and cytosolic staining H-scores (0-3 and H-score) as well as the combined H-scores according to the array and spot positions on the TMA. Also shown for each patient is the available follow-up survival data in months and DSS censor data (0= Disease specific death, 1= Censored) (not available= no number present).
