# Supplementary figures and images for: Endogenous Retroviral–K Envelope Is a Novel Tumor Antigen and Prognostic Indicator of Renal Cell Carcinoma
Source: Front Oncol. 2021 Apr 22;11:657187. doi: 10.3389/fonc.2021.657187 (PMC8100683; doi:10.3389/fonc.2021.657187)

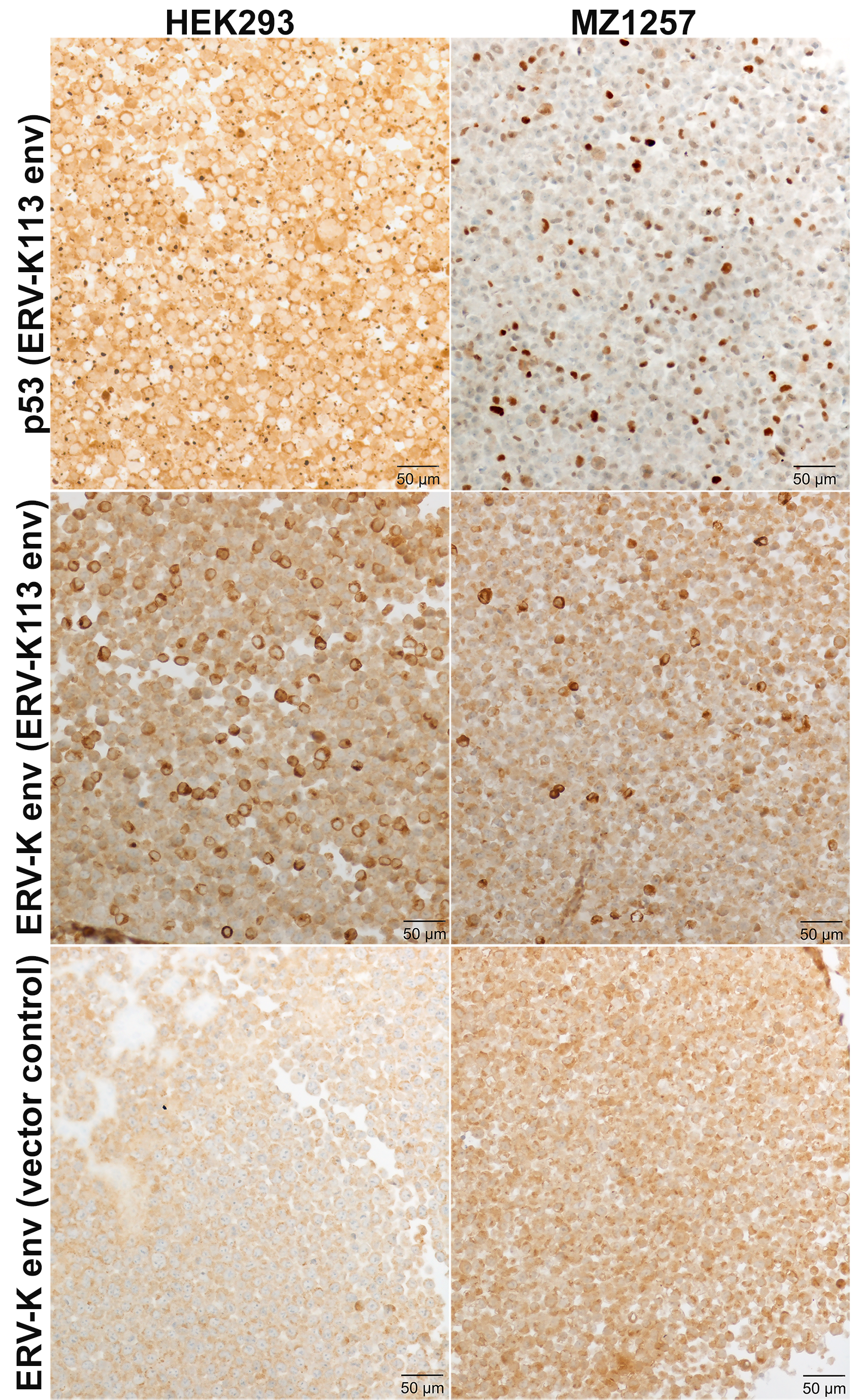

Supplement: Supplementary Figure 1 — Cytoblock of MZ1257RC and HEK293 cell sections hybridized with p53 and the ERV-K113 env antibody. Top left (HEK293) and right (MZ1257RC) photos show p53 localization hybridizing with a specific p53 antibody to cells harvested at 16 h post transfection with the overexpressing CMV vector containing a codon optimized ERV-K113 env gene. No difference was found with the untransfected control pcDNA3.1 vector (not shown). Middle panel left (HEK293) and right (MZ1257RC) photos show ERV-K113 env protein cellular localization hybridizing with a specific ERV-K113 env antibody to cells harvested at 16 hours post ERV-K113 env CMV vector transfection. Below left (HEK293) and right (MZ1257RC) photos show hybridization with a specific ERV-K113 env antibody to cells harvested at 16 hours post transfection with the control pcDNA3.1 CMV vector. A time gene expression kinetic showed the highest expression at 16 h (HEK293 2-ΔΔCt = 31,028.66; n= 2; MZ1257RC 2-ΔΔCt = 106,902.78; n= 2). Note that at 16 h the p53 wt HEK293 cell line was 3.44-fold lower in ERV-K113 env gene expression compared to the p53 mutant MZ1257RC cell line. This result was similar comparing both cell lines at the 24 h kinetic time point shown in Figure 6 . [file Image_1.tif]

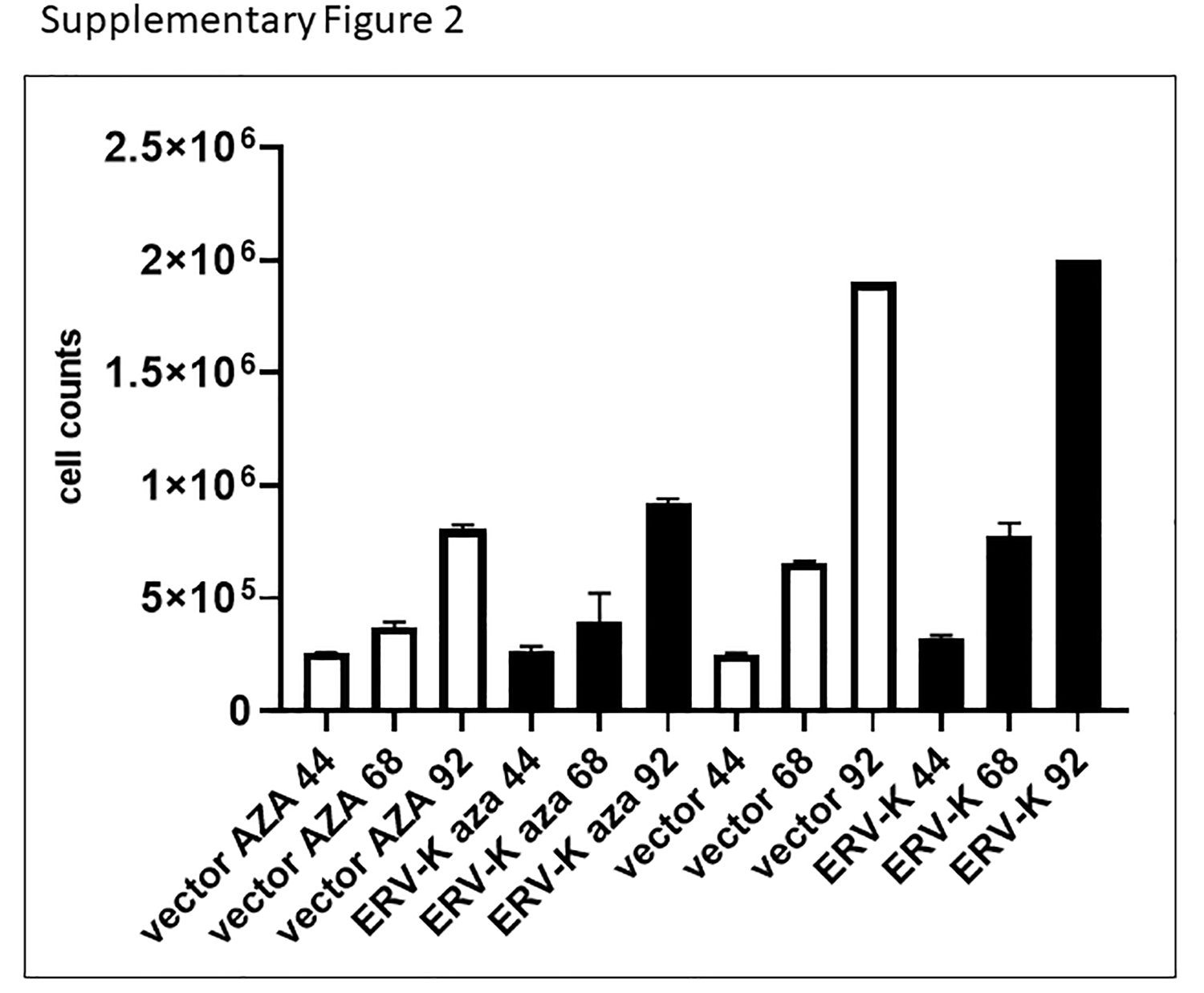

Supplement: Supplementary Figure 2 — HEK293 cell proliferation. Graph shows HEK293 cell proliferation (Y-axis = total cell counts) following transient transfection with the overexpressing CMV vector containing a codon optimized ERV-K113 env gene at 44 h, 68 h and 92 h post transfection in the presence or absence of Aza. (n= 2). [file Image_2.tif]
